# Importance of Spray–Wall Interaction and Post-Deposition Liquid Motion in the Transport and Delivery of Pharmaceutical Nasal Sprays

**DOI:** 10.3390/pharmaceutics14050956

**Published:** 2022-04-28

**Authors:** Arun V. Kolanjiyil, Ali Alfaifi, Ghali Aladwani, Laleh Golshahi, Worth Longest

**Affiliations:** 1 Department of Mechanical and Nuclear Engineering, Virginia Commonwealth University, Richmond, VA 23284, USA; avkolanjiyil@vcu.edu (A.V.K.); alfaifia@vcu.edu (A.A.); aladwanigh@vcu.edu (G.A.); lgolshahi@vcu.edu (L.G.); 2 Department of Pharmaceutics, Virginia Commonwealth University, Richmond, VA 23298, USA

**Keywords:** nasal spray, droplet impaction, spray–wall interaction, CFD, spray modeling, spray delivery, droplet deposition, liquid layer, surface film dynamics, liquid motion

## Abstract

Nasal sprays, which produce relatively large pharmaceutical droplets and have high momentum, are primarily used to deliver locally acting drugs to the nasal mucosa. Depending on spray pump administration conditions and insertion angles, nasal sprays may interact with the nasal surface in ways that creates complex droplet–wall interactions followed by significant liquid motion after initial wall contact. Additionally, liquid motion can occur after deposition as the spray liquid moves in bulk along the nasal surface. It is difficult or impossible to capture these conditions with commonly used computational fluid dynamics (CFD) models of spray droplet transport that typically employ a deposit-on-touch boundary condition. Hence, an updated CFD framework with a new spray–wall interaction (SWI) model in tandem with a post-deposition liquid motion (PDLM) model was developed and applied to evaluate nasal spray delivery for Flonase and Flonase Sensimist products. For both nasal spray products, CFD revealed significant effects of the spray momentum on surface liquid motion, as well as motion of the surface film due to airflow generated shear stress and gravity. With Flonase, these factors substantially influenced the final resting place of the liquid. For Flonase Sensimist, anterior and posterior liquid movements were approximately balanced over time. As a result, comparisons with concurrent in vitro experimental results were substantially improved for Flonase compared with the traditional deposit-on-touch boundary condition. The new SWI-PDLM model highlights the dynamicenvironment that occurs when a nasal spray interacts with a nasal wall surface and can be used to better understand the delivery of current nasal spray products as well as to develop new nasal drug delivery strategies with improved regional targeting.

## 1. Introduction

Nasal spray pumps are commonly used to deliver locally acting formulations to treat diseases and disorders such as allergic rhinitis, sinusitis, nasal polyposis and migraines [1,2,3,4]. Nasal spray pumps are popular due to their simplicity and ease of use, requiring no additional power or gas source [1,5,6,7,8,9]. For these products, the delivery efficiency of a nasal pump system can be affected by several factors including device design, formulation characteristics, patient-related factors such as inhalation conditions, and nasal geometry [7,10,11,12,13,14,15,16,17,18,19,20,21].

Understanding pharmaceutical spray device performance and quantifying drug delivery efficiency can help to improve drug delivery to the intended sites of action and may also assist in developing generic products through the assessment of bioequivalence [9,11,12,22,23,24,25]. In vivo, in vitro, and in silico testing can provide insights into device performance, spray plume characteristics and regional drug delivery within the nose [1,18,26,27,28,29,30]. In vivo testing, including radiolabeled imaging or plasma concentration measurements, may provide clinically relevant data [26,31]; however, in vivo experiments can be challenging and expensive. In vitro testing can be performed to extract device and formulation combination characteristics [17,21,32,33,34,35,36,37]. For evaluating generic nasal spray/aerosol systems that target local action, the *U.S. Food and Drug Administration (FDA) Draft Guidance on Bioavailability and Bioequivalence Studies for Nasal Aerosols and Nasal Sprays for Local Action* recommends in vitro spray testing and measurements of spray metrics, such as the single actuation content through container life, droplet size distribution, drug in small particles/droplet, spray pattern, plume geometry, and priming/repriming [38]. In vitro testing using realistic nasal airway geometries may also help to understand the geometrical factors influencing drug delivery to different regions of the nose [7,11,13,14,16,18,19,20,22,39,40,41,42,43]. In silico testing using computational fluid dynamics (CFD) simulations can provide detailed information on device performance and nasal deposition, and CFD models combined with pharmacokinetic models can provide additional information on systemic drug plasma concentration, while limiting time and cost associated with the testing [5,9,24,25,44,45,46,47,48,49,50,51].

In vitro deposition measurements are typically performed using nasal airway models made of rapid prototyped plastics, or molded with silicone, glass, or metal [8,13,18,28,42,52,53,54]. A primary focus of these in vitro investigations is often estimating the amount of drug delivered to the nasal regions past the nasal valve, which is the intended site of action for most nasal sprays. Considering the high momentum flow of nasal spray droplets, the impaction of spray droplets on nasal surfaces is expected to produce spray–wall interaction dynamics and liquid motion after impaction [9,55,56]. Furthermore, spray–wall interaction dynamics and post-deposition liquid motion may largely control the amount of drug delivered to specific nasal regions.

Spray impaction dynamics on a planar surface have been extensively investigated using computational and experimental tools. Impaction of droplets on solid and liquid surfaces leads to a variety of physical phenomena, such as spreading, droplet rebound, or splashing, which play a critical role in controlling the accuracy and efficiency of many mechanical systems [57]. These accompanying physical phenomena are highly complex with a multitude of interdependent factors influencing the final outcome [55,58]. There have been a number of recent experimental investigations into spray–wall interactions, because of the relevance to many engineering applications including fuel injection engines, spray cooling, spray coating, ice accretion/erosion on aircraft wings and inkjet printing [59,60]. Despite this previous work, the physics of spray–wall interactions continues to be a quickly developing and evolving field that is highly complex and not yet fully understood [61].

Only limited information is currently available regarding nasal spray–wall interaction and post-deposition liquid motion dynamics on nasal surfaces. The formation of a dynamic liquid layer after spray impaction on the nasal surface is expected for nasal sprays due to liquid cohesion and spray momentum leading to bulk motion that is enhanced by inhalation flow and gravitational force. Recently, Sosnowisky et al. [56] reported deposition of a nasal spray on pediatric nasal airway models and post-deposition liquid motion with inhalation flow during in vitro testing. In this in vitro study, the liquid spreading was determined using water-sensitive gel, which highlighted post-deposition liquid motion over time. Si et al. [50] computationally demonstrated nasal spray drug delivery to the upper nasal regions using gravity-driven translocation of the deposited spray liquid for a subject in a head-down position relative to gravity. Similarly, Inthavong et al. [62] computationally showed liquid motion on the nasal surface during nasal saline irrigation using a squeeze bottle. In a subsequent study, the authors investigated the effects of head tilt on liquid motion and its impact on wall stress using the computational Volume of Fluid approach [63,64,65]. As shown by Rygg et al. [44,45,66], Shang et al. [67,68] and Chari et al. [48], mucociliary clearance of the mucus layer on the nasal surface can lead to further increase in spray liquid motion, but over a longer time period. In view of in vitro testing of nasal sprays, spray–wall interaction with post-impaction liquid motion are principal factors of interest that may impact the regional distribution of drugs within the nose. In contrast, mucociliary clearance is not a factor determining the final regional nasal drug distribution during in vitro testing. Currently, there is only limited information available on spray–wall interaction dynamics and the post-deposition liquid motion of nasal sprays and, hence, further investigations are needed to better understand these complex phenomena.

Previous studies from our group developed approaches to capture two-way momentum coupling for nasal sprays but did not explore the associated effects of spray–wall interaction and post-deposition liquid motion [9,49]. In these studies, the spray deposition on the nasal surface was predicted using the two-way coupled Eulerian-Lagrangian model. The nasal surface was divided into anterior and posterior regions with a vertical slicing plane based on the nasal turbinates. However, in-house experiments showed that spray–wall interaction and post-impaction liquid motion can become predominant depending on the type of spray pump, spray nozzle positioning, liquid formulation properties, administration conditions, and nasal geometry. Furthermore, computational modeling difficulty increased when the anterior–posterior cut plane was closer to the anterior nasal valve in a more physiologically correct angled position [69]. It was observed that the liquid nasal spray formed a surface liquid film layer and flowed from the anterior to posterior region, which only required the deposited liquid to move by several millimeters. It is difficult or impossible to model these conditions with the typical standalone droplet transport model, which employs a deposit-on-touch boundary condition.

The objective of this study was to develop a new spray–wall interaction (SWI) model in conjunction with a model of post-deposition liquid motion (PDLM) on the nasal surface to simulate these physical phenomena that are known to occur during nasal spray drug delivery, and thereby produce a more realistic CFD approach. The SWI-PDLM model developed in this study is intended to capture in vitro nasal spray test conditions and is compared with corresponding in vitro data. As a result, mucociliary clearance effects that occur in vivo over longer time scales, as previously simulated by Rygg et al. [44,45,66], Shang et al. [67,68] and Chari et al. [48] were not included. It is anticipated that in many nasal spray delivery cases, the spray–wall interaction and post-deposition liquid motion largely control the final anterior to posterior distribution of the nasal spray delivery.

## 2. Materials and Methods

In the following section, a brief literature review of the physical phenomena of spray–wall interaction and short-term post-deposition liquid motion that are relevant to the delivery of nasal spray products are provided. Details of the SWI and PDLM models used in this study, followed by details on the nasal models, simulation set-up and in vitro test setting for model validations are provided in the subsequent sections.

### 2.1. Literature Review of Studies Evaluating Spray–Wall Interaction and Post-Deposition Liquid Motion

**Figure 1:**
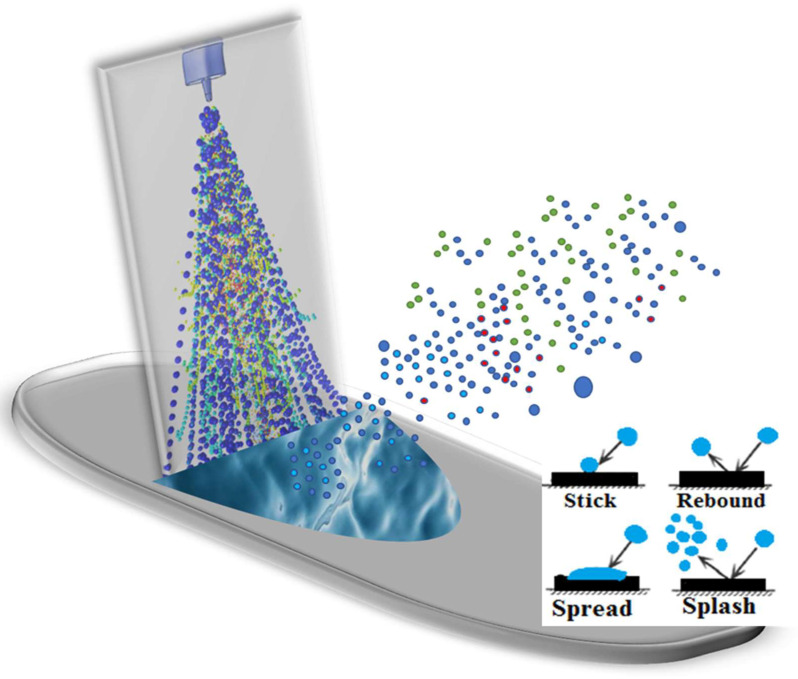
Graphical representation of liquid spray interaction with a wall surface at the point of impaction (colors are representative of a polydisperse spray droplet distribution).

Based on recent droplet–wall interaction experimental observations, major impaction regime outcomes (occurring below the boiling point of the liquid) are sticking, rebounding, spreading, and splashing (Figure 1) [70,71,72]. Droplets stick when the impaction energy is low, rebound when the impaction energy is moderate, spread when the impaction energy is moderate to high, and splash when the impaction energy is high [61,73]. Droplets that stick or spread on the wall form a liquid layer on the surface (Figure 1) [74]. These droplet–wall interactions and liquid layer dynamics are interactive and fully coupled; thus, both influence the fate of the droplets [59]. The final outcome of the droplet–wall interaction is mainly decided by the droplet impaction energy, the droplet liquid properties, and the solid material/surface conditions [59,75,76,77]. Specifically, factors including impingement velocity and angle, droplet size, and liquid properties such as liquid density, viscosity and surface tension influence the spray–wall interaction [60,77]. Additionally, other factors such as surface wall roughness, solid–liquid–gas interfacial energy, liquid contact angle, and presence of liquid on the surface also play a critical role in determining the fate of the impacting spray droplets [72,78].

Of the different spray–wall interactions, splashing is considered to be the most complex and diverse regime [55]. Splashing has been shown to be significantly affected by the spray droplet and wall properties [79,80]. The impaction energy and the impaction angle have positive correlations with spray droplet splashing dynamics [59,81]. Recent experimental investigations of droplets impacting on surfaces have indicated that the splashing mechanism is heavily influenced by surface roughness and the presence of a liquid layer on the impacting surface [79]. The surface roughness of the impacting surface has been shown to increase splashing behavior, while the presence of a liquid layer on the surface has been shown to suppress splashing [82]. The splashing behavior is vastly different on a dry surface compared to a wet surface with a liquid layer [79]. Depending on the thickness of the liquid layer on the impacting surface, spray–wall interaction can be again classified as thin film, liquid film, shallow pool and deep pool interaction [61,76]. The splashing behavior is distinct in these regimes and it is mainly controlled by the thickness of the liquid layer [83].

A number of recent computational studies have put forward spray–wall interaction models that can predict regime transitions based on experimental observations. Bai and Gosman [74,84] developed a phenomenological model to predict the spray–wall impaction while accounting for the different spray impaction regimes. In this model, the regime transition was modeled using correlations of Weber number and Laplace number derived from experimental observations [72]. The post-deposition characteristics were modeled by considering the mass, momentum and energy conservation before and after impaction [74,84]. O’Rourke and Amsden [85] developed a spray–wall interaction model together with a Lagrangian film model to simulate the absorption of the impacting droplets into the liquid film. In a later publication, O’Rourke and Amsden [86] improved the model by adding the splash regime transition based on the experimental observations. Han and Xu [87] further improved the O’Rourke and Amsden model by accounting for spray droplet spread and rebound. Stanton and Rutland [88] developed a spray–wall interaction model together with the film formation model based on experimental observations. In this model, the splashing threshold was estimated based on a splash parameter identified by the experimental investigations of Yarin and Weiss [89] and Mundo et al. [71]. A majority of these investigations are based on single droplet impaction. However, as shown in Yarin and Weiss [89], multi-droplet impaction studies are more suitable to define spray–wall interaction of spray impaction. In addition, most of these spray–wall interaction models are limited in addressing the influence of surface roughness and the presence of a liquid layer on the surface. Recently, a number of experimental investigations have focused on these situations and highlighted the corresponding spray–wall interaction dynamics [55,59,61]. In order to realistically simulate the spray–wall interaction dynamics for pharmaceutical nasal spray products, a new model considering all these specific conditions is needed. Hence, the new SWI model accounting for all relevant specific impaction conditions was developed in the current study.

### 2.2. Spray–Wall Interaction (SWI) Model

**Figure 2:**
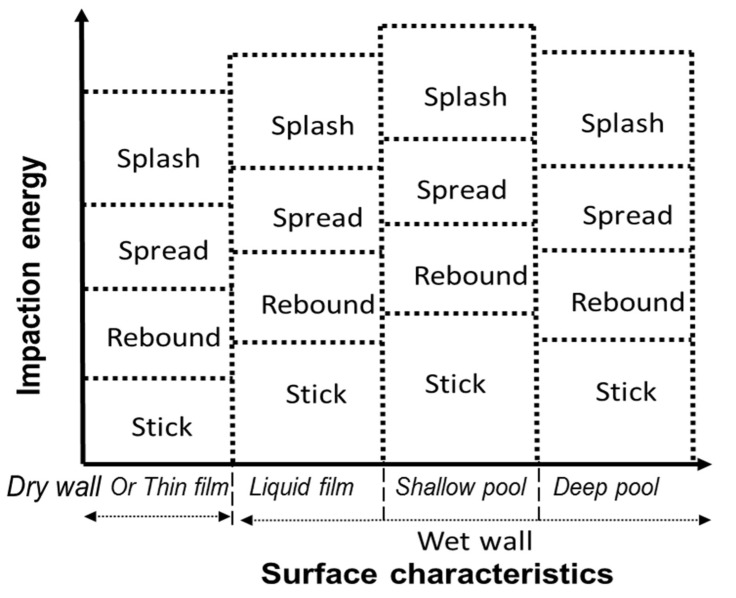
Spray–wall interaction model schematic diagram illustrating the different impaction and surface regimes.

Nasal spray droplets hitting a nasal airway surface can stick, rebound, spread or splash (Figure 1). Correspondingly, spray impaction conditions were captured using a new SWI model, as shown in Figure 2, and the impingement of droplets on the wall was transformed to stick, spread, splash, or rebound regime conditions. In addition to the typical spray impaction regimes, to account for the specific conditions that were mentioned in the last section, the current SWI model was developed by building upon the recent spray–wall models [72,88] based on recent experimental findings [58,59,60,72], as described in detail below.

Based on the physics involved in the spray–wall interaction, the dynamics of droplet impaction were described by various dimensionless numbers including the Weber number (We =$\;\frac{\rho dV^{2}}{\sigma }$), Reynolds number (Re = $\frac{\rho dV}{\mu }$) and Ohnesorge number (Oh = $\frac{\mu }{\sqrt{\rho \sigma d}}$), where *ρ*, *µ* and σ are the formulation density, viscosity and surface tension; *d* is the droplet diameter and *V* is the droplet impaction velocity normal to the surface. Two other dimensionless parameters that are important in deciding the impaction dynamics are dimensionless surface roughness ($R_{a}^{*}=\frac{R_{a}}{d}$) and dimensionless film height ($h^{*}=\frac{h}{d}$), where *R_a_* is the surface roughness and *h* is the liquid surface layer thickness. The impaction regime boundaries for each droplet–wall interaction were calculated in terms of these impaction parameters. A representative SWI model is shown in Figure 2. After incorporating the recent experimental observations [71,76,77,82,90], the current model accounts for the effects of surface roughness, liquid–wall adhesion and the presence of a liquid layer on the wall surface and the layer thickness.

Following the experimental observations by Tropea and Marengo [75], the spray–wall interaction can be again classified depending on the thickness/height of the liquid layer on the wall surface into a dry wall or thin film, liquid film, shallow pool, and deep pool regimes (Figure 2 and Table 1 list the parameter values which separate each regime). Other researchers have also noted similar classification of surfaces based on thickness/height of the liquid layer on the wall surface [76,79]. Considering that most of the rapid prototyped material surfaces used in nasal spray testing with in vitro nasal replicas are hydrophilic (contact angle < 90°), the following surface and impaction regimes have been incorporated into the SWI model. Table 1 lists the criteria determining the surface and impaction regimes based on the impaction parameters.

**Dry wall or thin film:** In this surface regime, the liquid layer is either not present or the liquid layer thickness is less than 3$\;R_{a}^{*}$^0.16^, where $R_{a}^{*}$ is the dimensionless surface roughness parameter. When the spray droplets impact the wall surface in this regime, the surface roughness strongly influences the spray–wall interaction [75]. Following the experimental observations that droplets with low impaction energy stick to the surface [73], the sticking regime was assumed for We ≤ 1 and the rebounding regime was assumed when We ≤ 4. Cossali et al. [90] proposed transition criteria between deposition and splash in terms of a parameter combining Weber and Ohnersorge numbers, i.e., the *K*-factor (*Kf = WeOh^−^*^0.4^), by curve fitting the experimental results of droplet splashing on surfaces with varying surface roughness (listed in Table 1). The *K*-factor parameter takes into account both viscous and surface tension effects while determining the spread–splash transition regime. The *K*-factor parameter was shown to be more suitable for capturing the droplet splashing behavior [71]. The *K*-factor relation derived in Cossali et al. [90] was used for defining the spread–splash regime transition.

**Liquid film:** In this surface regime, the effect of surface roughness on spray–wall interaction becomes weaker and the presence of the liquid layer on the surface starts to influence the interaction [75]. Based on the transition criteria proposed by Bai et al. [72], considering the effects of neighboring impacting droplets, a droplet was considered to stick for We ≤ 2 and a droplet was considered to rebound for We ≤ 20. The proposed sticking limit is in agreement with the experimental observations from Liang et al. [91] and Chen et al. [78]. Recent experimental investigations by Chen et al. [92] showed that the rebound characteristics of a droplet impacting on an inclined surface with a liquid layer can be related to the Weber number estimated using the wall-normal velocity. Results from Chen et al. [92] indicated a rebound limit of approximately We = 10. However, these results are based on single droplet impaction and, hence, the result from Bai et al. [72] was more suitable to define droplet rebound for spray impaction. The spread-splash regime transition was defined (Table 1) based on the experimental observations of Rioboo et al. [80], Cossali et al. [90] and Motzkus et al. [93].

**Shallow pool:** In this surface regime, the spray–wall interaction is mainly dependent on the thickness of the liquid layer on the surface. The transition criteria proposed by Bai et al. [72] were used for determining the stick and rebound regimes. The spread–splash regime transition was defined by an empirical relation correlating liquid film and shallow pool spread–splash regime transition values, following the inverse correlation proposed by Kalantari and Tropea [94] (Table 1).

**Deep pool:** In this surface regime, the spray–wall interaction is not affected by the surface roughness or the thickness of the liquid layer on the surface. The transition criteria proposed by Bai et al. [72] were used for determining the stick and rebound regimes. The spread–splash regime transition was defined by an asymptotic value for the *K*-factor corresponding to a critical Weber number proposed for spread–splash transition in a deep liquid layer [94] (Table 1).

After determining the spray–wall interaction regimes, the post-deposition droplet mass and velocity and the corresponding mass and momentum transfer were estimated based on specific impaction conditions as detailed below.

**Stick**: In this regime, the impacting droplet adhered to the surface.

**Rebound**: In this regime, the impacting droplet was assumed to reflect from the surface. The rebounding droplet velocity was set based on restitution coefficients derived from experimental observations [72]. The tangential and normal components of the rebounding velocity were defined as

(1) $$V_{r\;t}\;=\;\frac{5}{7}V_{p\;t}$$

V_r n_ = e_n_V_p n_ (2)

where V_p t_ and V_p n_ are the tangential and normal components of the impacting droplet and e_n_ is the normal restitution coefficient defined as

e_n_ = 0.993 − 1.76 Θ + 1.56 Θ^2^ − 0.49 Θ^3^ (3)

where Θ is the impingement angle (in radians) measured from the wall surface.

**Spread**: In this regime, the impacting droplet was assumed to merge with the liquid layer upon impact. The direction and velocity resulting from the coalescence were set using a wall-jet model [95].

**Splash**: In this regime, the droplet breaks into multiple droplets after impaction. During splashing, a fraction of the impacting droplet mass splashes into daughter droplets and the remaining fraction of the droplet mass adheres to the impacting surface or the liquid layer on the surface. Bai et al. [72] assumed a random distribution between 0.2 and 0.8 for dry wall and 0.2 and 1.1 for wet wall conditions. Based on experimental observations of splashing droplets by Yarin and Weiss [89] and Kalantari and Tropea [76], the mass fraction of the splashing droplet was defined as a random value evenly distributed between 0.2 to 1. The mass fraction of splashed droplet may go above 1 based on the liberation of additional mass from the surface in high momentum impaction [76]; however, to avoid numerical instability, a maximum splashing mass fraction limit of 1 was assumed. The droplet diameter distribution, velocity distribution and direction of the splashed droplets were randomly sampled from the experimentally obtained distribution functions of Mundo et al. [71].

### 2.3. Post-Deposition Liquid Motion (PDLM) Model

The liquid motion model solves the short-term post-impaction mass and momentum of the impinging droplets in an Eulerian framework and works in tandem with the spray–wall interaction model. The model is based on the lubrication theory of thin film layer dynamics and it is solved only on wall surface cells (pseudo 3–D). The conservation of mass and momentum of the liquid flow on the surface can be described as

(4) $$\frac{\partial \rho h}{\partial t}+\nabla_{s}.\rho h\bar{V}=\dot{m_{s}}$$

(5) $$\frac{\partial \rho h}{\partial t}+\nabla_{s}.\left[\rho h\bar{V}\bar{V}+{\vec{D}}_{v}\right]=-h\;\nabla_{s}\;\left[\;P_{gas}+P_{\vec{g_{n}}}+P_{\sigma }\;\right]+\rho h{\vec{g}}_{\tau }+\frac{3}{2}\;{\vec{\tau }}_{fs}-\frac{3\mu \;}{h}\vec{\;V}+\tau_{\theta w\;}+\dot{q_{s}}$$

where ρ is the liquid density, h the liquid layer height, $\nabla_{s}\;$the surface gradient operator, $\bar{V}\;$the mean liquid layer velocity, $\dot{m_{s}}\;$the mass source per unit wall area due to droplet collection, ${\vec{D}}_{v\;}$ the tensor denoting the differential advection term computed on the basis of the quadratic liquid layer velocity profile, and $\dot{q_{s}}\;$the momentum transfer during droplet collection on the surface. $P_{gas}$ captures the effects of gas–flow pressure, $P_{\vec{g_{n}}}\;$captures the effects of the gravity component normal to the wall surface (known as spreading), and $P_{\sigma }\;$captures the effects of surface tension based on the liquid layer film thickness curvature. The second term on the right hand side of Equation (5) represents the effect of gravity in the direction parallel to the liquid film, while the third and fourth terms represent the net viscous shear force on the air–liquid and liquid–wall interfaces, based on the quadratic velocity profile representation. The fifth term captures the effect of partial wetting of the surface by liquid, which is modeled as the surface force due to liquid surface tension and contact angle. The partial wetting can be modeled as

(6) $$\tau_{\theta w\;}=\beta \sigma \;\left[\;1-cos\;\theta_{w}\right]\nabla_{s}\;w$$

where σ is the film surface tension, β is an empirical parameter to account for discrepancies between the model and observed film behavior, and w represents the film wetted area fraction. The wetted area fraction takes a value of zero in dry regions and a value of 1 in wet regions. The film contact angle $\theta_{w}\;$is sampled randomly from a Gaussian distribution over the wall film characterized by the mean contact angle $\theta_{m}\;$and its standard deviation [96].

### 2.4. Nasal Airway Model

**Figure 3:**
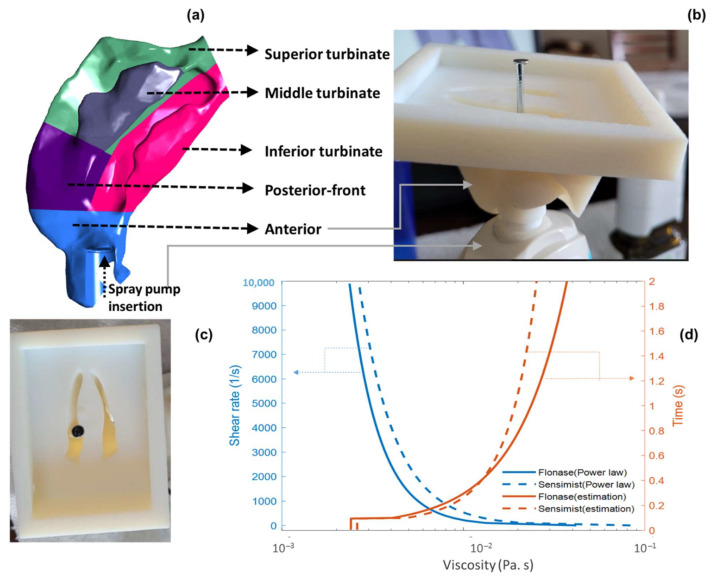
(**a**). Medium deposition model (7L) divided into specific nasal regions with Flonase spray pump inserted into the anterior nasal cavity. (**b**) Front and (**c**) top view of Flonase Sensimist spray pump inserted into the anterior part of the nasal model and metal pin showing the spray nozzle axis at the boundary with the posterior nose. (**d**) Viscous shear thinning and relaxation profile of Flonase and Flonase Sensimist formulation viscosity.

The nasal airway geometry used in this study was selected from an in-house set of 20 adult healthy nasal geometries (40 nasal cavities) that were extracted from computer tomography (CT) scans as part of a larger study to identify a range of representative nasal models based on nasal spray deposition [13]. The representative nasal airway was selected from the nasal models based on medium posterior spray deposition [13], i.e., the Medium Deposition or M nasal model. The selected model was Subject 7′s left nasal cavity, who was a 35-year-old male [69]. The associated nasal surface area and volume values were 34,579 mm^2^ and 61,435 mm^3^, respectively [69]. The CT image reconstructed nasal geometry was exported as stereolithographic (STL) formats. The STL nasal airway geometry file was then converted to computer-aided design (CAD)-accessible versions for developing the CFD model by skin-overlaying with large polygonal patches consisting of b-splined surfaces [49,97]. The nasal geometry was divided into anterior and posterior regions based on the location of the nasal valve (Figure 3a) [69]. The posterior region was again divided into posterior–front, inferior, middle, superior, and pharynx regions based on the turbinate locations (based on manual segmentation) (Figure 3a).

### 2.5. In Vitro Experimental Setup and Spray Delivery Measurements

In this study, two commercially available locally acting glucocorticosteroid nasal sprays, Flonase^®^ (active ingredient—fluticasone propionate) and Flonase^®^ Sensimist™ (active ingredient—fluticasone furoate) (Glaxo-SmithKline Consumer Healthcare, Warren, NJ), were selected as representative products. In vitro spray deposition on the nasal surface was measured for both sprays. For the in vitro deposition measurements, the Flonase Sensimist spray pump was hand-actuated and Flonase spray pump was actuated (with a force of 7.2 kgf) using the MightyRunt^®^ actuation station (InnovaSystems, Inc., Moorestown, NJ, USA), keeping the spray bottles in the vertical direction (following spray pump “instructions for use,” which recommends tilting the head forward slightly while keeping the bottle upright), as shown in Figure 3a [13]. The sprays were actuated at the beginning of a recommended “*gently sniffing*” inhalation flow [13,49,53], which was generated using a downstream breathing simulator (ASL 5000; IngMar Medical, Pittsburgh, PA, USA). The 3D printed nasal models were fabricated with high clarity rigid plastic (Accura ClearVue with standard finish), utilizing 3D Systems Quickparts printing (3D Systems, Valencia, CA, USA) [9,13,41]. The regional spray delivery percentages were defined as the fraction of the drug mass deposited in specific nasal regions divided by the total deposited drug mass in the entire model (×100). Anterior and posterior surface depositions were measured for Flonase and Flonase Sensimist, while sub-regional posterior surface depositions were measured only for Flonase. In vitro experimental setups and procedures for measuring the Flonase and Flonase Sensimist spray deposition in the nasal models are described further in previous publications [9,12,13,41].

The Medium nasal airway geometry with specific Flonase and Flonase Sensimist insertion conditions was used in this study (Figure 3a shows Flonase insertion in the CFD model and Figure 3b,c shows Flonase Sensimist insertion setup in the in vitro model). The in vitro nasal models were fitted with a spray adapter/holder ring with a thickness of approximately 1 mm placed in the anterior nose region. Specifically, the spray pump holder ring was used to position the spray pump at a known and controllable location, angle, and insertion depth [49]. The spray pumps (Flonase and Flonase Sensimist) were inserted into the left nasal cavity and aligned with the approximate center of the downstream nasal valve. The spray pump holder ring in the in vitro model and the corresponding CAD model were used as a reference to locate the spray pump and its nozzle orifice in the CFD model [98]. The volume of the spray pump holder ring and spray pump was subtracted from the nasal volume in the CFD model to simulate the presence of both. The spray pump insertion conditions were rechecked for any difference in the in vitro model after insertion through the holder ring into the 3D printed nasal model and consistently the spray pump position and the alignment were corrected in the computational model for both spray pump insertions. This was done by image analysis and visual inspection of the spray pump inserted into the 3D printed in vitro model. Images were taken after inserting the spray pumps into the 3D printed nasal models. Spray pump position inside the holder ring was measured to correct the insertion length in the CFD model. The spray pump axis inside the 3D printed nasal model was marked using a metal pin, as shown in Figure 3b,c. The alignment and the position of the pin with respect to the INV cut plane were used to correct the spray pump insertion condition in the CFD model. This step ensured correctly matched pump axis and location in both the in vitro and the CFD models. Specifically, the Flonase spray pump insertion condition consisted of 15.7 mm insertion depth from the nostril plane, head angle of 55.3°, and a 34° coronal angle. The Flonase Sensimist spray pump insertion condition consisted of 12 mm insertion depth from the nostril plane, head angle of 59°, and a 37° coronal angle. The head and coronal angles are the administration angles [13,49]. The head angle captures the head tilt with respect to the spray bottle and the coronal angle captures the spray pump axis with respect to the nasal septum. Insertion depth indicates the length of the spray pump inserted into the anterior nasal part [49].

### 2.6. CFD Simulation Setup with Specific Spray Parameters

To reduce the computational time and resources required for two-way coupled spray simulations, a momentum transfer approach was recently introduced in our previous studies [9,49]. The complete modeling framework to simulate Flonase and Flonase Sensimist spray impaction and deposition in the nasal models consisted of the momentum transfer spray-droplet transport model in combination with the SWI-PDLM model to simulate the spray–wall interaction and track the flow of liquid layers on the nasal surface after impaction (Figure 1). In the momentum transfer approach, the spray droplet momentum (two-way coupling) was simulated by transferring the momentum sources from a simplified geometry to the nasal model [49]. With the computational geometry and nasal CFD model, comparative analyses of spray delivery in specific nasal regions were performed to validate the SWI-PDLM model results. Additionally, results from the SWI-PDLM modeling framework were also compared with a standalone quasi two-way coupled framework to illustrate the influence of spray–wall interaction and post-deposition liquid motion. In the quasi two-way coupled framework, the spray droplets were simulated using the quasi two-way coupled model following the momentum-jet steady-state condition [98]. Different spray transport and deposition modeling approaches used in this study are listed in Table 2. Further details on the quasi two-way coupled and the momentum transfer spray transport and deposition modeling approaches are provided in our previous work [9,49].

Following the experimental procedure, sprays were actuated into the nasal cavity in conjunction with nasal inhalation flow (at the beginning of the flow cycle). Complete spray simulation consisted of two spray actuation cycles with an approximate ~2 s cycle period per actuation resulting in a total simulation time of ~4 s. [9,13]. The sprays were actuated at the beginning of the recommended “*gently sniffing*” inhalation flow pattern, which consists of an accelerating phase up to T = 0.4 s, with a peak flowrate of 20.2 L/min, followed by a decelerating phase until T = ~2 s [13,49,53]. The transient inhalation flow profile is plotted in the results section and the supplementary videos. CFD model-predicted deposition fractions in the anterior and posterior regions were calculated based on deposition in the entire model and the tracheal outlet escape fraction. The outlet escaped mass fraction was put into the posterior deposition fraction to allow reporting consistent with the experiments [49].

The initial and the boundary conditions of the CFD model were based on Flonase and Flonase Sensimist spray properties. Specifically, the spray droplets were injected slightly above the nozzle orifice (approximately 0.25 mm), following the spray cone projection, to ensure all injected droplets were initialized within the flow domain and to provide better solver stability. Droplets with specific diameters were injected with the measured polydisperse droplet size distributions (spray droplet diameter distribution previously reported in Ref. [49]), mean values of cone angle (28° for Flonase and 35° for Flonase Sensimist), spray mass flow rate (Flonase—shot weight 94.5 mg, spray duration 95 ms, density 1050 kg/m^3^ and Flonase Sensimist—shot weight 56.3 mg, spray duration 50 ms, density 960 kg/m^3^) and spray velocity (Flonase mean velocity 14.5 m/s and Flonase Sensimist mean velocity 14.4 m/s) [9,13,41]. A mass flow (inhalation flow) condition at the nostril inlet and a zero-gauge pressure condition at the nasal outlet were specified. Following the momentum transfer approach, the spray was actuated into the nasal flow domain with spray momentum transferred into the nasal model. The spray droplets were injected with an initial spray velocity assuming a turbulent velocity profile and in vitro measured droplet size distribution, specific to each spray under consideration. Further details on the model inputs are provided in our previous study [9,49].

Both spray formulations were noted to have shear-dependent viscosity. The apparent viscosity of each spray formulation was measured using a cone plate rheometer (Toolmaster™ (Physica MCR 301 TruGap™ Ready, Anton Paar)) [13]. During nasal spray actuation, the liquid spray formulation experiences shear rates on the order of 10^5^ s^−1^. Due to the limited range of shear that can be applied in the experimental methodology, a power law model (Ostwald-de Waele model) was used (by curve fitting measured viscosity vs. shear rate values) to estimate the liquid apparent viscosity at higher shear rates as shown in Figure 3d. Formulation viscosities for both sprays at high shear rates of 10^5^ s^−1^ and above were constant and approximately the same. Hence, in the SWI-PDLM simulations, during the spray actuation and the initial wall interaction, the liquid viscosity was considered to be at a shear rate of 10^5^ s^−1^. After the spray actuation and wall interaction, spray liquid experiences shear thickening with the reduction in the applied shear. The viscous relaxation profile, considering thixotropic (time-dependent) effects, was approximated based on the measured formulation viscosities, experimental observations, and the recent experimental viscosity measurements of Kippax et al. [99], in which the shear thickening of a nasal spray formulation was observed to rapidly increase the liquid viscosity by an order of magnitude in the first second when the applied shear was suddenly removed and then to slowly increase afterward. Similar behaviors for nasal spray formulations have been noted in other recent studies [35,100,101]. Based on in-house observations and preliminary simulations, Flonase Sensimist was assumed to have a slightly slower relaxation than Flonase. A sensitivity study was also performed to investigate the effects of the relaxation profile on nasal deposition by interchanging the relaxation profiles. The effect of inhalation flow shear on liquid viscosity was not directly applied considering that during peak flow the liquid is already at a relatively high shear-low viscosity phase from the spray actuation.

A pendant drop method was used with a drop shape analyzer (DSA25E, Krüss, Germany) to measure the formulation surface tension for the two sprays [13]. The formulation surface tension for Flonase was 0.044 ± 0.8 N/m and Flonase Sensimist was 0.042 ± 0.6 N/m. The surface roughness of the 3D printed material (Accura ClearVue with standard finish) was measured using a contact profilometer (Mitutoyo SJ-410). The average surface roughness was measured to be 1.51 ± 0.056 µm. The surface wetting effects were included in the model by measuring the static contact angle on the 3D printed material surface using a ramé-hart Goniometer and DROPimage software. The static contact angles for Flonase and Flonase Sensimist liquid formulations on the 3D printed material surface were 53.5° ± 2.3° and 71.9° ± 2.82°, respectively. Contact angle hysteresis was not considered in this study; however, variability in contact angle measurements was applied by randomly sampling from a Gaussian distribution based on the measured mean contact angle and standard deviation for each formulation.

### 2.7. Numerical Methodology

CFD simulations were performed using ANSYS Fluent 2021.R1 (ANSYS Inc., Canonsburg, PA, USA). As noted in our previous study, considering the high maximum Reynolds number value and the large recirculatory flow patterns of nasal flows near the nasal spray liquid jet, the computations were performed using a transient incompressible low-Reynolds number (LRN) k-ω turbulence flow solver [49]. The solution was deemed to be converged when all mass and momentum residuals dropped by at least three orders of magnitude and did not change with further iterations. A time step size of 1 × 10^−7^ s was used for solving the liquid motion model during the active spray period and the droplets were injected with a time step of 1 × 10^−2^ s. The flow solver time step was incrementally increased after the active spray period of ~0.1 s while confirming solution convergence. During this time, the liquid motion solver time step was also proportionally increased. For both sprays considered in this study, a total simulation time of 4 s was deemed sufficient after observing that a further increase in simulation time by 0.1 s resulted in less than 0.5% change in the regional spray mass. In-house ANSYS Fluent user-defined functions (UDFs) were used to apply variable time steps for the flow solver. Separate UDFs were used to apply variable viscosity and ascertain multi spray liquid fractions following a homogenous mixture model formalism. UDFs were used to transfer the momentum source terms from the simplified model to the nasal model and specify momentum source terms [49]. UDFs were also used to identify droplet impingement conditions and specify the droplet spray wall interaction model. A separate UDF was used to specify the droplet rebound model, as the built-in function resulted in unrealistic droplet trajectories due to over estimation of the deviation angle or the azimuthal angle from the incident direction. Additional UDFs were used to specify the recommended “*gently sniffing*” inhalation flow pattern and to initialize the solver domain, which effectively identified cells near the spray nozzle within the spray cone volume and initialized the flow variables only in these cells [49]. Following the established meshing parameters for nasal spray modeling, nasal geometries were discretized with ~2 million polyhedral elements per nasal cavity, excluding the nasopharyngeal region, together with near-wall prism elements and a refined mesh in the spray nozzle tip vicinity. For simulating one cycle of nasal spray transport in conjunction with the nasal inhalation flow, the SWI-PDLM model simulation took ~24 h on a 55 core Linux system. Further details on the governing equations, solver settings and additional details of the CFD procedure are available in Kolanjiyil et al. [9,49].

## 3. Results

### 3.1. Spray–Wall Interaction and Post-Deposition Liquid Motion on the Nasal Surface

**Figure 4:**
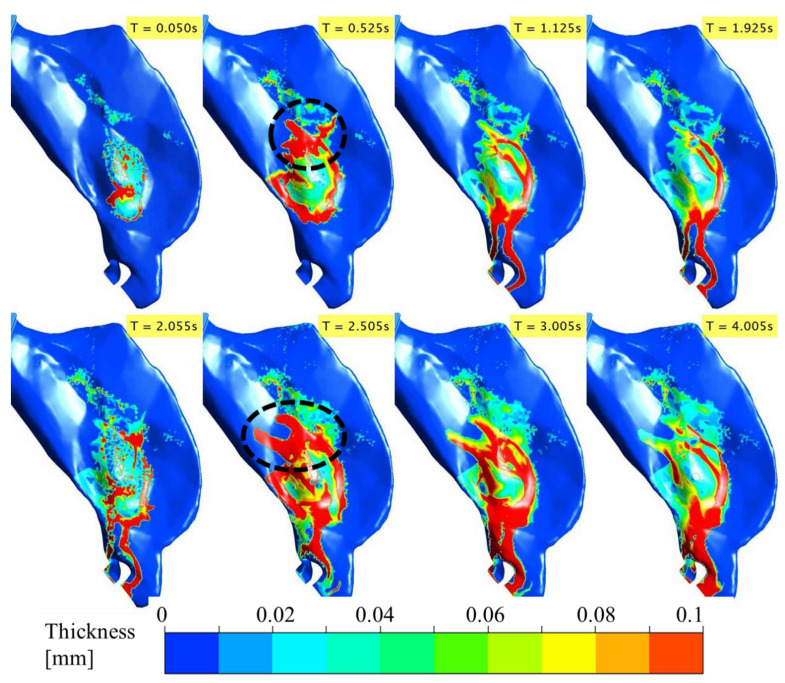
Evolution of the deposited Flonase liquid on the nasal airway surface during the two spray actuation cycles at specific time points noted in the upper right corner of each sub-figure panel. (Dotted circle represents the high liquid mass concentration in the posterior region).

**Figure 5:**
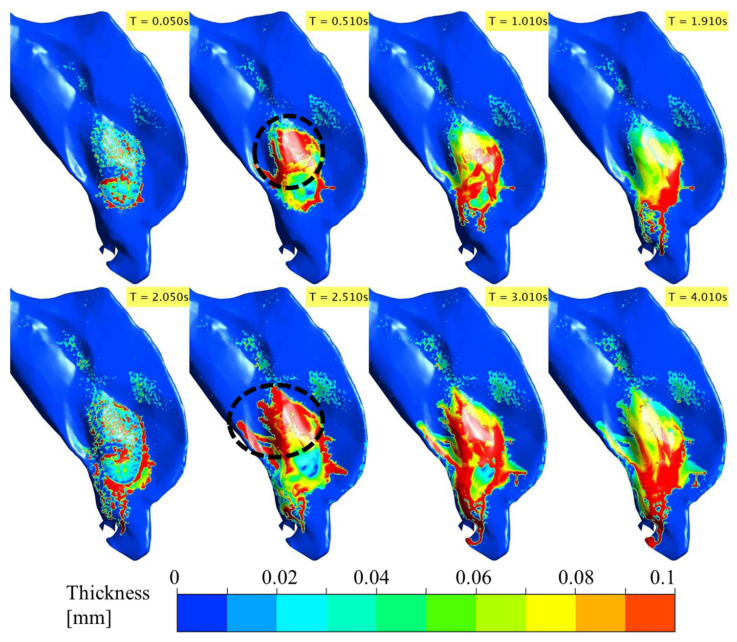
Evolution of the deposited Flonase Sensimist liquid on the nasal airway surface during the two spray actuation cycles at specific time points noted in the upper right corner of each sub-figure panel. (Dotted circle represents the high liquid mass concentration in the posterior region).

Flonase and Flonase Sensimist spray droplet transport, impaction, and post-impaction liquid motion on the nasal surface were simulated using the SWI-PDLM model. Figure 4 and Figure 5 illustrate the evolution of Flonase and Flonase Sensimist spray liquid on the nasal airway surface (at specific time points), respectively. In these figures, the maximum contour value of the liquid thickness was limited to 1 × 10^−4^ m to delineate the regions containing appreciable spray mass. The maximum thickness of the liquid on the nasal surface over the two spray cycles was less than 1 mm for both sprays, except at the edge of the anterior nasal wall close to the nostril opening where spray liquid was allowed to collect and flow out at the wall edge, where it was classified as drip consistent with the experiments. The results from the spray droplet–wall interaction simulation indicated that the initial spray impingement resulted in a thin initial wall film of approximately 0.05 mm average thickness. As the spray droplet impingement continued, the momentum transfer from the successive spray impingement broke the initial wall film. With the continuing passage of spray time, successive droplets impinged on the nasal surface leading to repeated film breakage and liquid movement on the nasal surface from the initial location (as noticed at time T = 0.05 s). Flonase had a relatively narrow cone angle compared to Flonase Sensimist, which resulted in less dispersed deposition. As seen at T = 0.05 s, the wet surface area for the Flonase spray was relatively smaller when compared to the wet surface area for Flonase Sensimist. Furthermore, at T = 0.05 s, Flonase spray resulted in a smooth and connected liquid film on the nasal surface while the Flonase Sensimist spray resulted in sporadic liquid film patches. These differences could be mainly attributed to the differences in the spray liquid mass emitted at this time point (based on liquid mass flowrate), as well as differences in the formulation characteristics, spray properties and spray insertion conditions. Interestingly, both sprays resulted in higher liquid film concentration in specific nasal surface locations, which had inward (concave) surface curvatures.

As the spray injection time continued, the liquid motion on the nasal surface became more prevalent. After the first spray stopped (T = 0.095 s for Flonase and 0.05 s for Flonase Sensimist), the deposited spray-liquid moved along the surface in the direction of the gravitational vector, following the nasal surface curvature. The effects of increasing inhalation flow towards the nasopharyngeal region on the spray-liquid motion were more evident due to the shear effect from the inhalation flow. Since the spray was actuated in conjunction with nasal inhalation flow, the spray-liquid was pushed upward (to the back into the posterior nasal region) against gravity by the inhalation flow (as seen at T = 0.5 s). The inhalation flow peaked at ~0.4 s and a majority of the spray-liquid mass was pushed to the posterior region from the initial location during the acceleration phase of the inhalation (the dotted circle represents the high liquid concentration in the posterior nasal region). During the deceleration phase of the inhalation flow, the liquid moved downwards (towards the anterior part of the nasal cavity) in the direction of the gravitational force. However, wet surfaces were still noticed with a thin layer of liquid coating on the upper posterior regions of deposition. With the increase in time, bulk Flonase liquid motion formed a “finger” like flow pattern, resulting in a conduit for bulk flow towards the anterior tip of the nasal cavity (0.5 s < T < 2 s).

The second spray was also actuated in conjunction with a second inhalation cycle after ~2 s from the start of the first spray. With the second spray, there was an increase in the liquid bulk flow because of the increase in the overall liquid mass on the nasal surface (Figure 4 and Figure 5 and Supplemental Videos S1 and S2). During the second active spray period, liquid drippage motion continued through the finger-like liquid bridges. During the acceleration phase of the inhalation flow and second spray cycle (at T = 2.5 s), the upward motion of the spray-liquid against gravity was prominent and moved substantial liquid to the posterior nasal region. Once the inhalation flow subsided, the spray-liquid moved along the surface in the direction of the gravitational vector (dripping down) and tended to accumulate in regions of the nasal surfaces with inward curvatures. Compared to the first spray cycle, the liquid drippage increased in the second cycle (T = 3–4 s). Flonase liquid motion on the nasal surface was more prominent compared to that of Flonase Sensimist primarily due to higher injected spray liquid mass, as well as the differences in the formulation characteristics, spray properties and spray insertion conditions.

### 3.2. Comparison of Model Predicted Drug Delivery with In Vitro Measurements

**Figure 6:**
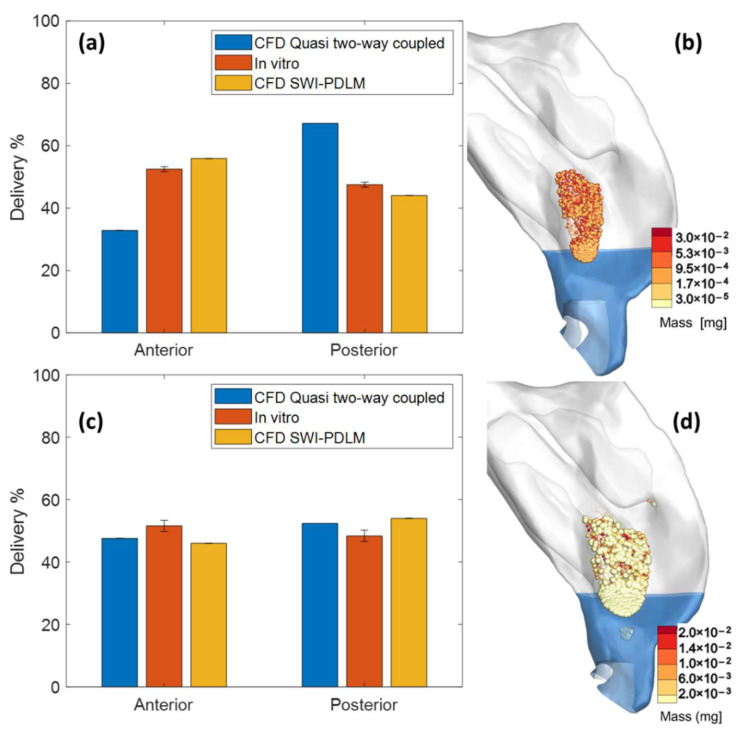
Comparison of the Flonase (**a**) and Flonase Sensimist (**c**) spray deposition prediction by the quasi two-way coupled Lagrangian model and the SWI-PDLM model with the in vitro measurements. Flonase (**b**) and Flonase Sensimist (**d**) spray droplet deposition locations predicted by the quasi two-way coupled Lagrangian model.

The deposition predicted by the SWI-PDLM model was validated by comparing the liquid mass in the anterior and the posterior regions of the nasal cavity with in vitro measurements. Figure 6a,c shows the comparison of Flonase and Flonase Sensimist spray deposition prediction by the SWI-PDLM model (predicted liquid mass at the end of 4 s simulation) and the quasi two-way coupled Lagrangian model (deposit-on-touch) with the in vitro measurements. The nasal spray deposition predictions were close to the 10% relative error margin with the new modeling approaches. Table 3 lists the comparison and the relative error estimation with respect to the in vitro measurements. Flonase spray deposition prediction by the SWI-PDLM model (6.6% and 7.3% relative error in anterior and posterior regions) showed closer agreement with the in vitro measurements compared to the quasi two-way coupled Lagrangian model (38.1% and 42.1% relative error in anterior and posterior regions), which predicted a lower anterior deposition and higher posterior deposition. Flonase Sensimist spray deposition predictions by both the SWI-PDLM model (10.8% and 11.5% relative error in anterior and posterior regions) and the quasi two-way coupled Lagrangian model (7.8% and 8.3% relative error in anterior and posterior regions) were similar and showed close agreement with the in vitro measurements.

Figure 6b,d shows the Flonase and Flonase Sensimist spray droplet deposition locations predicted by the quasi two-way coupled Lagrangian model. In the quasi two-way coupled model, the droplets were assumed to deposit at initial wall contact and lose all momentum (deposit-on-touch), hence, the spray–wall dynamics of rebound, spread and splash with subsequent liquid motion were not modeled. As shown by the SWI-PDLM model, the nasal spray deposition can be classified into spray–wall interaction phenomenon and liquid motion phenomenon. During spray–wall interaction, a portion of the impacting spray deposits at the impingement location, while the remaining liquid mass either spreads, rebounds, or splashes. After the spray–wall interaction, a portion of the liquid moves along the surface and crosses regional surface boundaries based on the driving forces of shear stress from inhalation flow and gravity. Considering that Flonase had a higher liquid motion along the nasal surface towards the anterior part of the nasal cavity, the SWI-PDLM model showed an increase in the liquid mass in the anterior region (by approximately 20%) and a corresponding decrease in the posterior region (by approximately 20%). However, the quasi two-way coupled Lagrangian model did not account for this and, hence, under-predicted the anterior deposition and over-predicted the posterior deposition. For Flonase Sensimist, the spray–wall interaction and the post-deposition liquid motion introduced competing effects with relatively equivalent magnitude; hence, both the SWI-PDLM model and the quasi two-way coupled Lagrangian model showed a similar end result.

### 3.3. Evolution of Spray-Liquid Mass in Specific Regions of the Nasal Airway Surface

**Figure 7:**
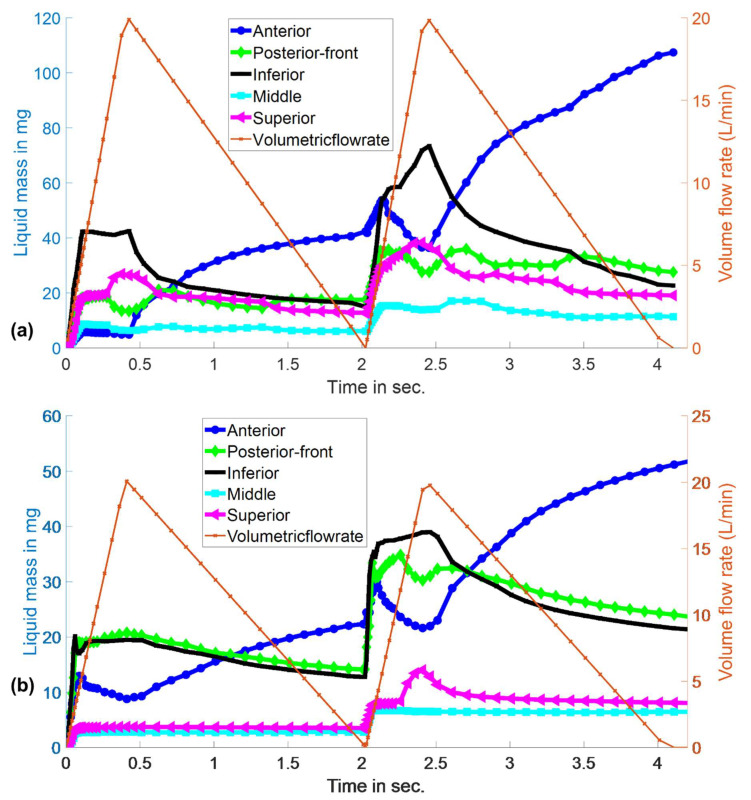
Evolution of (**a**) Flonase and (**b**) Flonase Sensimist spray-liquid mass in specific regions of the nasal airway surface.

Figure 7a,b shows the time evolution of Flonase and Flonase Sensimist liquid mass in specific regions of the nasal airway surface (Supplemental Videos S1 and S2). The evolution of the spray-liquid mass on specific nasal surfaces was transient in nature during the active spray period and the active inhalation flow period. Over time, the liquid motion reached a stable state with the reduction of the inhalation flow effects and accumulation of the liquid in the inward (concave) nasal surfaces and the anterior nasal surfaces. During the initial active spray period (T < ~0.1 s), spray-liquid masses within the inferior and posterior–front regions were higher compared to the anterior, middle and superior regions. The superior region received more spray-liquid with Flonase than Flonase Sensimist. However, after the active spray period, the spray liquid motion reached a pseudo-steady state due to the balance of forces from the gravitational pull to the anterior side and the inhalation flow to the back of the nose. With the peak inhalation flow (at T ~ 0.4 s) more liquid was pushed to the back of the nose due to the shear force from the inhalation air. As the inhalation flow subsided, liquid flowed down from the posterior–front and inferior regions into the anterior region, leading to an increase in the liquid mass in the anterior region. During the second spray actuation cycle, all the nasal regions received additional spray liquid. **The droplet–wall interaction was visibly different because of the already existing liquid layer from the first spray actuation.** Similar flow behavior was noticed in the second cycle, in which liquid flowed to the back of the nose during peak inhalation flow and liquid flow to the anterior nose after the peak inhalation flow. The liquid mass flowing down from the inferior and posterior–front regions to the anterior region was higher during the second cycle. This was due to the increased mass accumulation and downward bulk flow along the gravitational direction from the nasal surface curvatures. Considering the large spray shot weight and the differences in the formulation characteristics including viscosity, surface tension and contact angle, spray properties and spray insertion conditions, Flonase had a higher post-impaction liquid motion compared to Flonase Sensimist.

**Figure 8:**
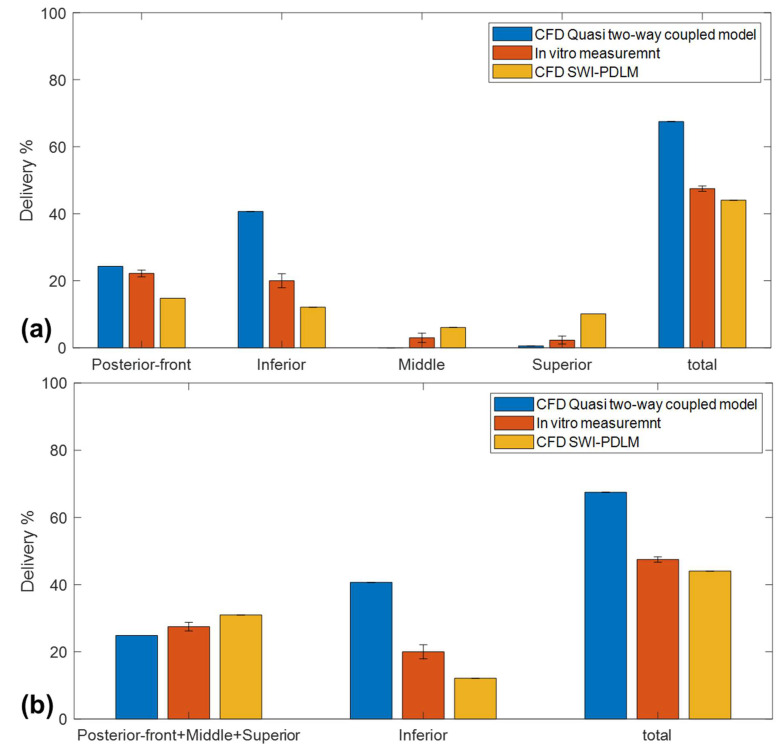
Comparison of the Flonase spray deposition in (**a**) specific posterior regions (**b**) in the upper and lower posterior regions predicted by the SWI-PDLM model and the quasi two-way coupled Lagrangian model with the in vitro measurements.

Flonase deposition in specific posterior nasal regions (posterior–front, inferior, middle and superior) was compared with the in vitro regional deposition measurements. Figure 8a shows the comparison of the Flonase spray deposition in specific posterior regions predicted by the SWI-PDLM model and the quasi two-way coupled Lagrangian model with the in vitro measurements. Overall, the regional deposition prediction by the SWI-PDLM model showed comparatively better agreement to the in vitro measurements. The quasi two-way coupled model showed better agreement only in the posterior–front, however, it over-predicted the deposition in the inferior region and under-predicted the deposition in the middle and superior regions. The SWI-PDLM model prediction showed under-prediction in the posterior–front region and over-prediction in the middle and superior regions.

**Figure 9:**
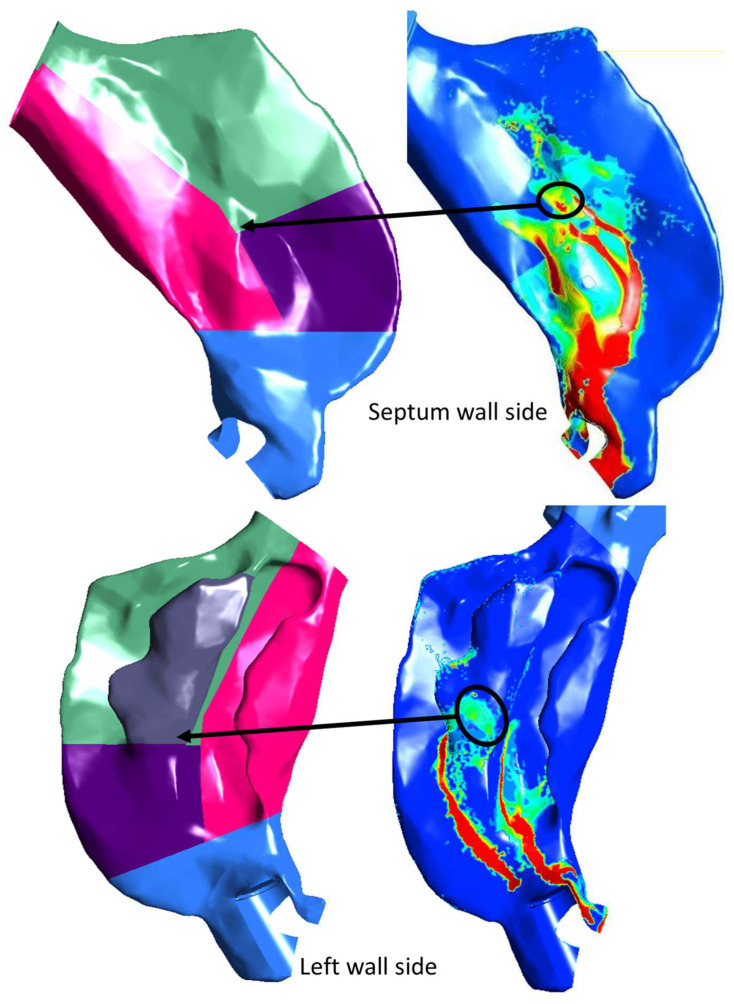
Specific posterior nasal regions and the corresponding spray liquid mass deposition predicted by the SWI-PDLM model on the nasal septum wall (upper image panel) and left-side outer wall (lower image panel).

Considering the mucociliary clearance pathway from the anterior part of the posterior region through the upper/middle and inferior regions into the nasopharyngeal region, the posterior region was regrouped into upper and lower regions. Figure 8b shows the vertical regional deposition comparison after regrouping the posterior regions into these upper and lower regions. The SWI-PDLM model predicted deposition in the upper region, which consists of the previous posterior–front, middle and superior regions, and showed better agreement with the in vitro measurements. On close inspection of the regional deposition prediction by the SWI-PDLM model for Flonase, as shown in Figure 9, the hot spot deposition locations (higher concentration of liquid mass) in the middle and superior regions were very close to the posterior–front boundary (less than 1 mm distance to the boundary).

## 4. Discussion

In this study, new models for simulating spray–wall interaction and post-deposition liquid motion of nasal sprays were developed. The simulation results provided insight into the physical mechanisms that occur during in vitro nasal spray testing using rapid prototyped in vitro nasal models. For the two spray pumps considered in this study, both sprays showed high spray–wall interaction, which resulted in droplet spreading, rebounding, and splashing. Furthermore, after deposition, droplets from both spray pumps formed liquid films and the liquid moved along the nasal surface due to both spray impaction momentum and inhalation flow. During the peak inhalation flow, the liquid motion was amplified by the shear flow resulting in liquid motion towards the back of the nose. Considering the large spray shot weight and the differences in the formulation characteristics including viscosity, surface tension and contact angle, spray properties and spray insertion conditions, Flonase had a higher post-impaction liquid motion compared to Flonase Sensimist. Hence, the SWI-PDLM model is more suitable for the prediction of spray deposition in these cases and can improve agreement with corresponding in vitro experimental results. Similar results have been observed experimentally by Sosnowski et al. [56] in a pediatric nasal airway model, where liquid formulations were observed to spread from the initial deposition sites to the back of the nose due to the inhalation flow.

In a previous study, the effect of nasal inhalation flow on spray droplet transport and deposition was found to be minimal, when the spray was actuated at the beginning of the inhalation cycle [49]. In the scenario when the spray is actuated at the beginning of the inhalation flow, the active spray period is shorter in comparison to the full inhalation cycle and, hence, the air flow that interacts with the spray droplet has a relatively low velocity. In this study, it was noted that even though the spray was actuated at the beginning of the flow, the post-deposition liquid motion was largely influenced by the inhalation flow. The liquid motion was particularly high during the peak inhalation air flow, resulting in more liquid flowing towards the back of the nose. This phenomenon of nasal spray drug delivery has been long-expected, but not previously observed with a CFD model to our knowledge.

Predictions of deposition in specific nasal regions were validated with in vitro deposition measurements. The regional spray deposition in the anterior and posterior regions predicted by the SWI-PDLM model showed overall closer agreement to the corresponding in vitro measurements. Flonase regional spray deposition predictions by the SWI-PDLM showed substantial improvement whereas the Flonase Sensimist showed a similar prediction trend compared to the standalone quasi two-way coupled spray deposition model. A recent investigation showed that the nasal spray carries significant momentum and during spray droplet transport, this spray momentum is exchanged with the surrounding gas field [9,49]. The resulting two-way coupled momentum exchange effect was noted to influence the nasal spray deposition pattern. For the Flonase Sensimist spray pump, the inclusion of two-way coupling was sufficient to improve regional agreement with in vitro data to a 10% relative error margin, which was not changed with the inclusion of SWI-PDLM modeling. However, for sprays with higher spray–wall interaction and post-deposition liquid motion, the SWI-PDLM model together with the two-way coupled spray transport model is needed, as noted in this study with the Flonase spray product to reduce relative errors in the CFD predictions.

The spray deposition predictions by the SWI-PDLM model for Flonase in the sub-posterior regions were found to have a reasonably close agreement with the in vitro measurements. When the posterior region was split into multiple smaller surfaces based on turbinates, the sub-regional surfaces had relatively smaller surface areas, and the liquid masses were shown to accumulate at the sub-regional boundaries. The agreement between the prediction and the in vitro measurement was improved when the surfaces were re-grouped to upper and lower posterior regions, considering mucociliary clearance from front to back of the nasal cavity [48,66,67,102].

Further improvements in the modeling approach such as applying specific spray formulation relaxation profiles, including micro-to-nano scale surface topology effects on the liquid motion, and including contact angle hysteresis effects could potentially improve the sub-posterior regional model predictions. Based on a preliminary sensitivity analysis by interchanging the relaxation profiles for Flonase and Flonase Sensimist, the deposition trend in the anterior and posterior regions varied by 3–4% in absolute difference and the maximum variation in the sub-posterior deposition was less than 1.5%. Apparent viscosity measurements and relaxation profiles at high shear rates using capillary rheometers, extensional viscosity measurements using extensional rheometers, or microfluidic rheometers could be used to supplement the model input with experimental data on the shear thickening behavior of nasal spray formulations after the sudden removal of high shear rates of order 10^5^ s^−1^ [35,103]. However, nasal spray formulations are typically blended with viscosity enhancing agents such as microcrystalline cellulose and carboxymethyl cellulose, and, hence, the viscosity of the formulation is dependent on shear stress history [35,100,101]. It should be noted that differences in spray pump shaking/priming procedure, rate of ramp-up and ramp-down, multiple spray pump actuation or applied shear during viscosity measurements could all influence the formulation viscosity and, as a result, the relaxation profile. Likewise, surface topology mapped in 3D using an atomic force microscope (AFM) or an optical surface profilometer could be used to improve the surface roughness measurements and surface topology effects [104]. Similarly, dynamic contact angle measurements could be used to improve the modeling of adhesive force and wetting effects and their influence on liquid motion while also accounting for the surface inhomogeneity, effects of surface roughness and surface curvature [105,106].

In this study, the nasal geometry used was the Medium Deposition model that featured median posterior delivery based on nasal spray deposition measurements in a larger data set of 40 nasal geometries [13]. Considering that the specific spray insertion captured deposition in the median range for adults, the spray is more aligned to the center of the anterior–posterior nasal wall boundaries. For nasal geometries with spray insertion resulted in direct and immediate impaction on the nasal surfaces, the effects of spray wall interaction and post-deposition liquid motion could become comparatively more pronounced. Hence, in a future study, different nasal airway models will be used to provide additional details on the effects of the SWI and PDLM. Future studies will also focus on mucus layer and mucociliary clearance effects, simulating in vivo conditions.

## 5. Conclusions

In this study, new SWI and PDLM models were developed using CFD simulations to understand the nasal spray–wall interaction and accompanying surface liquid layer dynamics that occur during experimental nasal spray testing using rapid prototyped models of nasal airway geometries. For both nasal spray products, time-resolved visualization of the surface liquid transport revealed a significant effect of the spray on the film followed by post-deposition liquid motion driven by the competing forces of airflow generated surface shear stress and gravity. For Flonase, these factors resulted in a significant shift in the final liquid resting position. For Flonase Sensimist, a significant liquid motion was also observed; however, factors moving the liquid to the posterior region were largely counteracted over time by factors moving liquid to the anterior nasal region such that the final resting place of the liquid was little changed. The SWI-PDLM model deposition predictions were validated with corresponding in vitro measurements. Overall, the SWI-PDLM model predictions had a closer agreement with the in vitro measurements compared to other previous nasal spray deposition modeling approaches. Specifically, when the SWI-PDLM model was used compared to the quasi two-way coupled model, the average relative error between the anterior and posterior regional spray deposition predictions and the in vitro measurements was reduced from 40.1% to 6.9% for Flonase. The relative errors for the SWI-PDLM model and the quasi two-way coupled model were similar for Flonase Sensimist. Based on the results from this study, depending on the spray pump administration and insertion angles, nasal spray actuation could lead to direct droplet impaction on the nasal airway surface at a short range. In these cases, droplet rebounding, spreading, and splashing followed by significant liquid motion after initial wall contact and the liquid motion after deposition along the nasal surface could become even more prominent. Hence, the need for SWI and PDLM models is more complex and likely dependent on a complex group of factors including the spray formulation, spray pump shot weight, priming and repriming conditions, number of shots, nasal geometry, positioning of the spay nozzle within the nose, surface properties of the in vitro model or in vivo airway surface, and point of initial spray–wall contact.

## Figures and Tables

**Table 1:** Spray–wall interaction (SWI) model: transition criteria for impaction regimes based on surface characteristics.

Surface Characteristics	Impaction Regime	Transition Condition	
Dry wall or thin film $(h^{*}\leq \;3R_{a}^{*}{}^{0.16}$)	Stick	We < 1	$\mathrm{where}\;K_{\mathrm{trans}}=649.0+\frac{3.76}{R_{a}^{*}{}^{0.63}}$
	Rebound	We < 4	
	Spread	K-f < K_trans_	
	Splash	K-f > K_trans_	
Liquid film $(3R_{a}^{*}{}^{0.16}<h^{*}\leq \;1$)	Stick	We < 2	where K_trans_ = $9582.0\;h^{*}{\;}^{0.51}\;$when $h^{*}$ ≤ 0.05 2100.0 + 2000.0$\;h^{*}{\;}^{1.44}$ when 0.05 < $h^{*}$ ≤ 1.0
	Rebound	We < 20	
	Spread	K-f < K_trans_	
	Splash	K-f > K_trans_	
Shallow pool $(1<\;h^{*}\leq \;2$)	Stick	We < 2	where K_trans_ = 4000.0$\;h^{*}{\;}^{-0.24}$
	Rebound	We < 20	
	Spread	K-f < K_trans_	
	Splash	K-f > K_trans_	
Deep pool $(h^{*}>2$)	Stick	We < 2	where K_trans_ = 3390.0
	Rebound	We < 20	
	Spread	K-f < K_trans_	
	Splash	K-f > K_trans_	

**Table 2:** Different spray transport and deposition modeling approaches used in this study.

Flonase and Flonase Sensimist Spray Droplet Transport and Deposition Models		
	Two-Way Coupled Euler–Lagrange Model + Spray–Wall Interaction + Post-Deposition Liquid Motion	Stand-Alone Two-Way Coupled Euler–Lagrange Model
Two-way coupling effect modeled using	‘Momentum transfer’ approach	‘Quasi two-way coupled’ approach
Droplet–wall interaction	SWI model	Deposit-on-touch
Liquid motion	PDLM model	Not modeled

**Table 3:** Comparison of CFD predicted spray deposition in specific nasal regions using the SWI-PDLM and the quasi two-way coupled models with the in vitro measurements.

	Flonase		Flonase Sensimist	
	Anterior (%)	Posterior (%)	Anterior (%)	Posterior (%)
CFD SWI-PDLM model	55.95	44.05	46.0	54.0
CFD quasi two-way coupled model (with deposit-on-touch droplet boundary condition)	32.50	67.50	47.6	52.4
in vitro	52.5 ± 0.8	47.5 ± 0.8	51.6 ± 1.8	48.4 ± 1.8
Relative error (SWI_PDLM model) (%)	6.6	7.3	10.8	11.5
Relative error (quasi two-way coupled model) (%)	38.1	42.1	7.8	8.3

## Data Availability

Data is contained within the article and the supplementary material.

## Supplementary Materials

The following supporting information can be downloaded at: https://www.mdpi.com/article/10.3390/pharmaceutics14050956/s1, Video S1. Evolution of Flonase spray-liquid mass in specific nasal regions. Video S2. Evolution of Flonase Sensimist spray-liquid mass in specific nasal regions.
